# Mechanical Properties and Performance of 3D-Printed Acrylonitrile Butadiene Styrene Reinforced with Carbon, Glass and Basalt Short Fibers

**DOI:** 10.3390/polym16081106

**Published:** 2024-04-16

**Authors:** Evgeniy Lobov, Ilia Vindokurov, Mikhail Tashkinov

**Affiliations:** Laboratory of Mechanics of Biocompatible Materials and Devices, Perm National Research Polytechnic University, 29 Komsomolsky Ave., Perm 614990, Russia

**Keywords:** 3D printing, composites, ABS, short fiber, mechanical properties, strength, fracture toughness

## Abstract

This paper presents the results of experimental investigation of the mechanical characteristics of 3D-printed acrylonitrile butadiene styrene (ABS) and its modifications reinforced with different types of short-fiber fillers: carbon, glass, and basalt. Elastic modulus, tensile and bending strength, as well as fracture toughness were determined in series of mechanical tests for samples produced with different manufacturing parameters, such as nozzle diameter and infill angle. It was found that the use of ABS filament reinforced with the short fibers can significantly improve the mechanical properties of 3D-printed devices when the infill angle is oriented along the vector of the applied load. In such a case, the elastic modulus and tensile strength can be increased by more than 1.7 and 1.5 times, respectively. The use of a larger nozzle diameter led to the growth of tensile strength by an average of 12.5%. When the macroscopic load is applied along the normal to the printed layers, the addition of short fibers does not give much gain in mechanical properties compared to pure ABS, which was confirmed by both standard tensile and fracture toughness tests. The surface of the fractured samples was examined using scanning electronic microscopy, which allowed us to make conclusions on the type of defects as well as on the level of adhesion between the polymeric matrix and different types of short fibers.

## 1. Introduction

Additively manufactured (AM) composites have attracted increased attention in the last decade due to their potential as functional components in high-demanding applications. Thanks to the existing capabilities of thermoplastics synthesis, short fibers (SFs) can be incorporated into traditional materials for 3D printing to improve their mechanical properties. Compared with conventional materials, such AM composites have shown increased physical and mechanical properties adaptable to a wide range of tasks [1,2,3,4,5,6]. 

Acrylonitrile butadiene styrene (ABS) is extensively used in additive manufacturing for its favorable balance of mechanical properties, ease of use, and affordability. However, inherent limitations such as low tensile strength, poor impact resistance, and susceptibility to warping under stress hinder its use in high demanding engineering applications. Short fibers, when added to ABS filament, offer several advantages that contribute to the improved mechanical properties and enhanced functionality of 3D-printed parts. These include increased tensile strength, stiffness, impact resistance, dimensional stability, heat, and chemical resistance.

**Figure 1:**
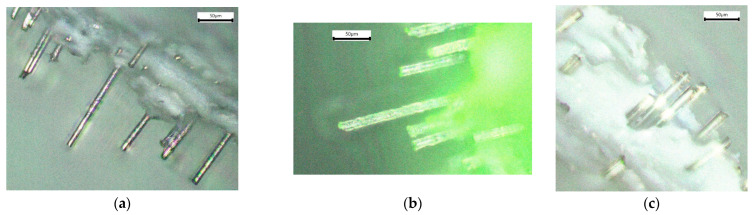
Optical microscopy images of short fibers in ABS matrix: (**a**) carbon, (**b**) glass, and (**c**) basalt.

This study investigates and compares the effectiveness of carbon (CF), glass (GF), and basalt (BF) short fibers (see Figure 1) as reinforcement agents in ABS filament for 3D printing. 

Each type of fiber offers unique properties and benefits, influencing the suitability of a material for specific applications. For instance, the addition of CF to ABS results in an exceptional strength-to-weight ratio and stiffness. These particles are lightweight, which helps to maintain the overall weight of the 3D-printed parts while still providing excellent strength. Moreover, CFs are electrically conductive, which can be advantageous for applications requiring electrical conductivity or static dissipation [3,7,8,9,10]. While CFs offer the most exceptional performance benefits, they are typically more expensive than other reinforcing agents, such as GF or BF. GFs, typically made of borosilicate glass, are valued for their cost-effectiveness, providing improved strength, stiffness, impact resistance, dimensional stability, temperature resistance, and chemical resistance [11,12,13,14,15]. BFs, which are derived from basalt rock through a process of melting and extrusion, offer a balance between performance and affordability, making them a compelling choice for many applications [16,17,18,19].

There are numerous studies reported in the literature in which SFs were blended with thermoplastic polymers, such as ABS and polylactide acid (PLA) [7,10,20,21,22,23,24,25]. A significant increase in tensile strength and elastic modulus was observed compared to the pure, unreinforced thermoplastic matrices. Thus, in [26], it was confirmed that the addition of short GF to ABS improved the strength of the material but reduced the flexibility of the polymer filament. A similar study was conducted with the addition of short CF in ABS [22]. O. Carneiro [27] found that the incorporation of GF into polypropylene increased the tensile modulus and strength by 30% and 40%, respectively. In the paper by F. Ning et al. [24], the effect of addition of CF on the mechanical properties of ABS polymer was studied. It was found that the tensile and flexural properties of the composite material increased in the range from 10% to 35% for different volumes of short CFs. Moreover, the effect of fiber content and length on the mechanical properties of ABS composite samples was evaluated. It was found that there is a certain limit for the increase in tensile strength that can be reached by adding a higher volume of fiber. Once this limit was reached, the tensile strength decreased to values similar to those of pure thermoplastic. It was observed that specimens made of longer CF showed higher tensile strength and elastic moduli compared to those made of shorter ones. Similar results are demonstrated in other related studies [22,28,29,30,31]. The tensile properties of 3D-printed samples of CF-reinforced ABS were studied in [3,8,10,26]. The interlayer mode-I fracture toughness was examined in [3] and [10] using a double cantilever beam (DCB) test.

The previous findings clearly show that the addition of SF to ABS increases its strength and elastic modulus. However, the increase in the mass fraction of short fibers in the polymer matrix is often associated with an accumulation of undesirable defects such as pores, which appear as a result of insufficient adhesion between the matrix and the inclusions. The predominant failure mechanisms in such composites are interfacial delamination, cracking of the matrix material, failure of the reinforcing fiber, and fiber pull-out [7,32]. The addition of a short fiber filler may as well impair the bonding quality of printing layers because of an increasing number of voids. Moreover, the interface between the SF and matrix, due to the large aspect ratio of the SF compared to the particle-type filler, may be weakened due to poor adhesion. Therefore, structure analysis at the micro-scale level is necessary to predict the onset and development of the fracture process. Tekinalp et al. [22] analyzed the relationship between the microstructure and mechanical properties of the 3D-printed SF-reinforced samples compared to the samples produced by the compression molding method. They discovered that 3D-printed composites exhibited relatively high porosity (20%), but the strength and modulus of elasticity in both materials were comparable. The difference between the two types of SF-reinforced samples was negligible, with the arrangement of fibers at an infill angle of 0–90°. In a study by Hou et al. [33], porosity was shown to have a significant influence on the mechanical properties of 3D-printed parts since the porosity rate increased with larger fiber contents, leading to the formation of larger pores. In addition, the distribution of pores at different fiber contents based on experimental data was observed. 

Despite the amount of work that has been already conducted in this field, the existing drawback is that, due to the specifics of AM technologies, all the results were obtained for different printing parameters using materials and equipment supplied by various manufacturers. Hence, there is a lack of a purely comparative analysis of influence of different types of short fibers embedded in the exact same ABS matrix on the effective mechanical properties, taking into account the control of the manufacturing process parameters.

The objective of this study is to perform a comparative analysis and investigate the impact of various types of SFs on the mechanical characteristics and properties of 3D-printed ABS, considering the variability in the manufacturing parameters, such as nozzle diameter and infill angle. New results regarding the stiffness and strength properties of SF-reinforced samples were obtained in a series of tensile and bending tests and compared with those of a conventional ABS material. Additionally, the surface of fractured samples was examined using scanning electronic microscopy (SEM). 

## 2. Materials and Methods

### 2.1. Manufacturing of Samples

The fused filament fabrication (FFF) AM technology was used for the production of samples. The pure ABS filament, as well as that with embedded CF, GF, and BF with a diameter of 1.75 mm, was supplied by REC (Moscow, Russia). Hereinafter, the following notation will be used for brevity: ABS + CF, ABS + GF, and ABS + BF will stand for ABS material reinforced with short carbon, glass, and basalt fiber, respectively.

**Figure 2:**
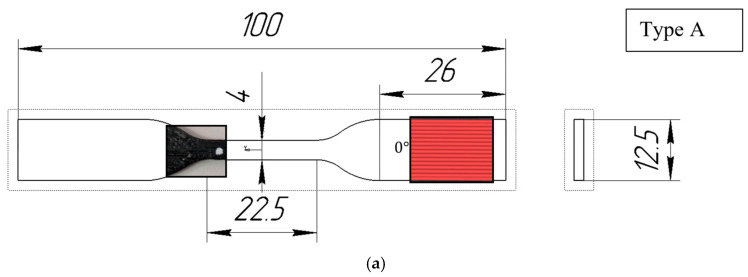

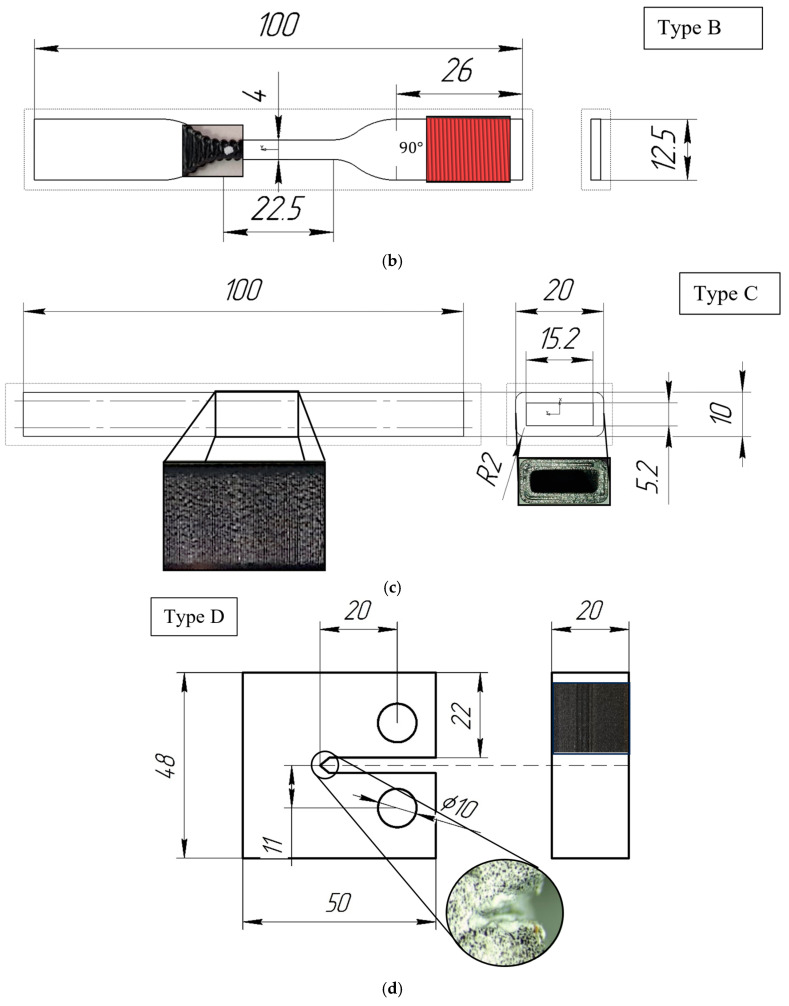
Geometry of test specimens: (**a**) Type A, (**b**) Type B, (**c**) Type C, and (**d**) Type D.

The tensile samples were designed in accordance with ISO 527-2:2012 standards [34] (Figure 2a,b). The samples for three-point bending tests were cylindrical rods (length 100 mm, width 20 mm, and height 10 mm) with an internal hole (length 100 mm, width 15.2 mm, and height 5.2 mm). The dimensions of the hollow part were chosen to ensure that the thickness of the walls could be evenly divided by the path widths produced by different nozzles during printing (Figure 2c).

For the production of samples, a Raise3D Pro2 Plus (Irvine, CA, USA) 3D printer was used. For the reinforced ABS samples, the following printing parameters were used: a layer thickness of 0.2 mm for a 0.4 mm nozzle and 0.4 mm for a 0.8 mm nozzle, a bed temperature of 110 °C, a nozzle temperature of 290 °C, a 100% rectilinear infill pattern, and a printing speed of 30 mm/s. These parameters conform to standard printing settings. For pure ABS, the bed temperature was 100 °C, and the nozzle temperature was 255 °C. All samples were printed in a closed chamber and naturally cooled to room temperature. The designation “Type A” corresponds to samples with a 0° infill angle, “Type B” corresponds to samples with a 90° infill angle, and “Type C” corresponds to hollow samples for three-point bending analysis. When printing Type A samples, a perimeter contour was used to secure the model. 

Compact tension (CT) samples (Figure 2d) were manufactured for the determination of fracture toughness parameters in accordance with ASTM D5045-14 [35].

### 2.2. Mechanical Testing

Tensile tests were conducted on a universal testing machine, Instron 68SC-5 (Glenview, IL, USA), equipped with a 5 kN load cell, at a constant loading rate of 0.5 mm/min, at ambient temperature (~20 °C). The servo drive displacement resolution was 0.0095 µm. A video extensometer, AVE2, was employed to measure deformations. Two points, equidistant from the midpoint, were marked on the surface of the specimens along the primal axis. The video extensometer measured the elongation of the samples by tracking the displacements of these points. The elastic properties under tension were obtained from the analysis of the elastic region of the stress–strain curve. Each specimen configuration underwent testing 15 times.

The parameter of resilience, which is the capacity of a material to absorb energy without undergoing permanent deformation or damage, can be represented by the area under the stress–strain curve formed by experimental stress and strain data points. The entire area under the curve is divided into a finite number of geometric figures (trapezoids), which are formed by pairs of stress and strain points from the experimental data. To determine the discrete area of each trapezoid, a standard formula was used:

(1) $$S=\frac{\sigma_{1}+\sigma_{2}}{2}\left(\varepsilon_{2}-\varepsilon_{1}\right)\;.$$

From a geometric perspective, this means that $\sigma_{1}$ and $\sigma_{2}$ represent the lengths of the bases of the trapezoid, while the difference $\left(\varepsilon_{2}-\varepsilon_{1}\right)$ represents the height of the trapezoid. To compute the total area under the curve, it is necessary to sum up all the values of the discrete areas.

Bending tests were conducted at a loading rate of 0.1 mm/min using a 500 N load cell. Each specimen configuration underwent testing at least 5 times to assess repeatability. The properties of the specimens under bending were extracted from the stress–strain curve

(2) $$\sigma =\frac{6Flh}{4(bh^{3}-b_{1}h_{1}^{3})},$$

where h is the height of the specimen, b is the width of the specimen, $h_{1}$ is the height of the inner part of the specimen, and $b_{1}$ is the width of the inner part of the specimen.

The fracture properties of the tensile specimens were evaluated in accordance with ASTM D5045-14 [35] standard for measuring the fracture toughness of polymers under flatwise plane strain. The crosshead speed of the Instron 68SC-5 universal testing machine was set to 1 mm/min. The critical stress intensity factor $K_{Ic}$ was determined using the formula

(3) $$K_{Ic}=\frac{P_{Q}}{BW^{1/2}}f\left(x\right),$$

where $P_{Q}$ is the applied load, B is the thickness of the specimen, W is the width of the specimen, a is the crack length, and x=a/W.

(4) $$f\left(x\right)=\frac{\left(2+x\right)\left(0.886+4.64x-13.32x^{2}+14.72x^{3}-5.6x^{4}\right)}{{\left(1-x\right)}^{\frac{3}{2}}}\;.$$

Additionally, the mode I critical strain energy release rate was determined according to the formula

(5) $$G_{Ic}=\frac{\left(1-\nu^{2}\right){K_{Ic}}^{2}}{E}$$

where E is the elastic modulus obtained in the fracture toughness test, ν is the Poisson’s ratio, and $K_{Ic}$ is the critical stress intensity factor. A VIC-3D Micro-DIC system (Correlated Solutions, Columbia, SC, USA) was used for measurements of strains. Prior to testing, a black-and-white speckle pattern was applied to the specimens with an airbrush. Images were obtained using two 5.0-megapixel cameras. The average strain value in the crack opening zone was evaluated using VIC-3D software. The fracture properties of the specimens were obtained from an analysis of the stress–strain curve.

**Figure 3:**
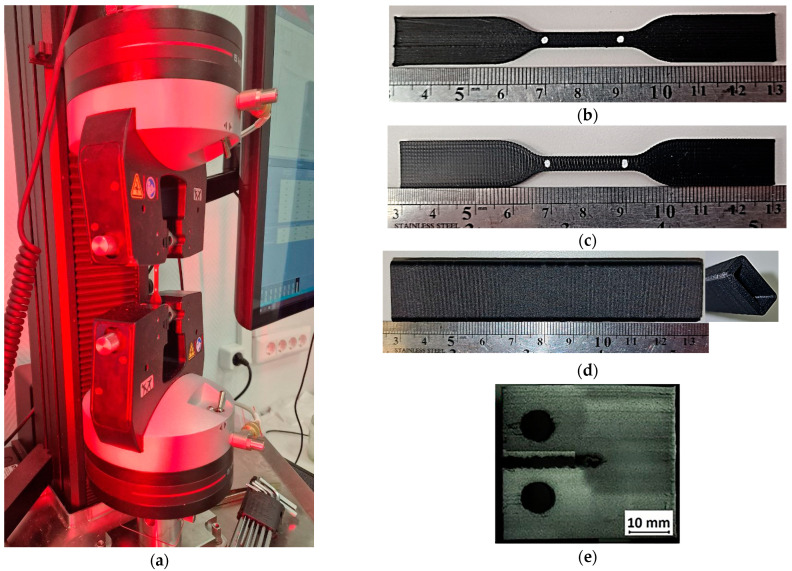
Testing set-up (**a**) and examples of 3D-printed specimens: (**b**) Type A, (**c**) Type B, (**d**) Type C, and (**e**) Type D.

The experimental set-ups, as well as some examples of the manufactured specimens of various types are presented in Figure 3.

### 2.3. Microtomography

The internal microstructural characteristics of the 3D-printed composites were investigated using tomographic imaging that was conducted for samples of filament reinforced with SF. A SkyScan 1272 micro-CT system (Billerica, MA, USA) was used. The imaging parameters were set as follows: X-ray tube voltage of 37 kV, current of 57 μA, voxel edge size (resolution) of 1 μm, exposure time of 3000 μs, sample rotation step of 0.1°, and complete 360° imaging with averaging over 4 frames. 

### 2.4. Scanning Electronic Microscopy

Surface analysis of the specimens was conducted using a COXEM EM-30 plus scanning electron microscope (Daejeon, Republic of Korea) at magnifications of 100×, 200×, 500×, and 1000×. Prior to morphological analysis, a thin layer of gold was sputtered onto the specimens to increase their electrical conductivity. An accelerating voltage of 15 kV was used. The analysis was performed using a secondary electron (SE) detector.

## 3. Results

### 3.1. Stiffness and Strength

#### 3.1.1. Tensile Properties

The values of elastic modulus and tensile strength, grouped by the SF type and nozzle diameter, are presented in Table 1. The abbreviated designation for the samples includes the nozzle diameter used. For example, “Type A + CF 04” denotes an ABS Type A specimen reinforced with short carbon fibers, printed using a 0.4 mm diameter nozzle.

**Figure 4:**
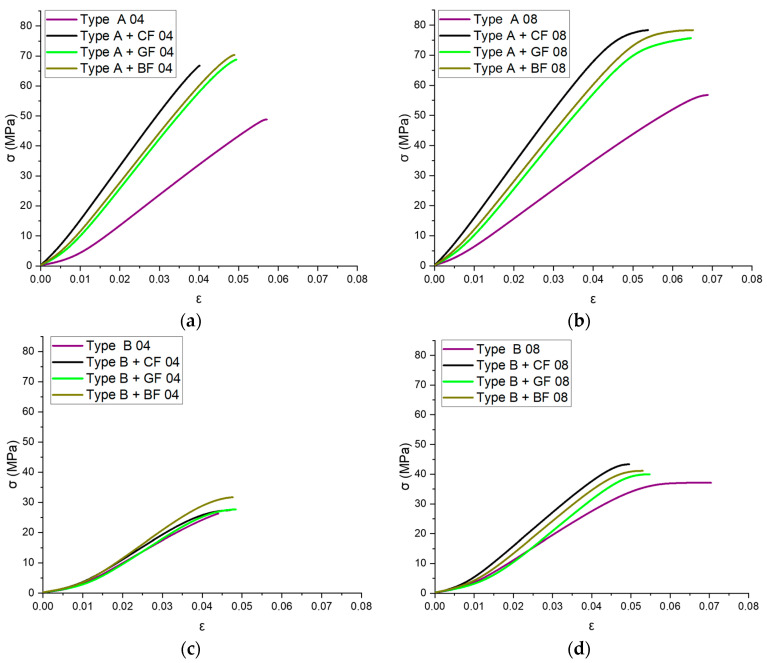
Stress–strain dependence for pure ABS and SF-reinforced ABS samples: (**a**) 0.4 mm nozzle, 0° infill angle, (**b**) 0.4 mm nozzle, 90° infill angle, (**c**) 0.8 mm nozzle, 0° infill angle, and (**d**) 0.8 mm nozzle, 90° infill angle.

The average stress–strain values obtained from nine tensile experiments are graphically represented as curves in Figure 4. The results are grouped according to parameters such as nozzle diameter and infill angle. All materials demonstrated nonlinear behavior within the load range specified.

#### 3.1.2. Fracture Toughness

The values of critical stress intensity factor $K_{Ic}$ and critical strain energy release rate $G_{Ic}$, as well as fracture strength obtained from the tensile testing of the CT specimens, are presented in Table 2.

**Figure 5:**
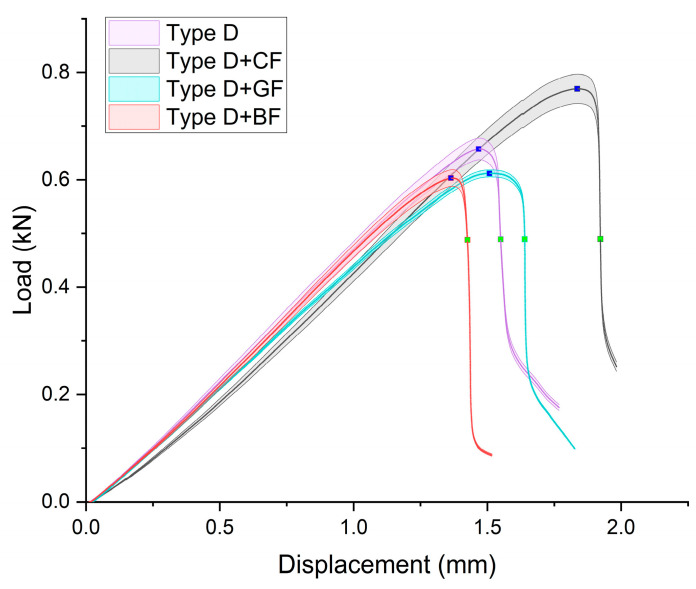
Load–displacement curves for Type D specimens. Blue dots correspond to initiation of the crack, and green dots indicate the load value for which images of the crack opening are shown in Table 3.

The averaged “load–displacement” curves with standard deviation for Type D specimens are presented in Figure 5. The curves had good repeatability both within the samples made of the same material as well as for materials with different types of SFs. The specimen filled with CF had a notably larger elastic zone, followed by ABS + BF, ABS + GF, and pure ABS.

The VIC-3D images of strain fields at the surface of Type D samples captured at the beginning of crack propagation, as well as at the crack opening stage corresponding to the applied tensile force value of 490 N, are provided in Table 3. These events are marked on the curves in Figure 5 with blue and green dots, respectively. The force value required for crack initiation for the pure ABS is 651.9 N, which is 18% less than that of the CF-reinforced material and 6% and 8% higher than that of the materials reinforced with GF and BF, respectively. 

**Figure 6:**
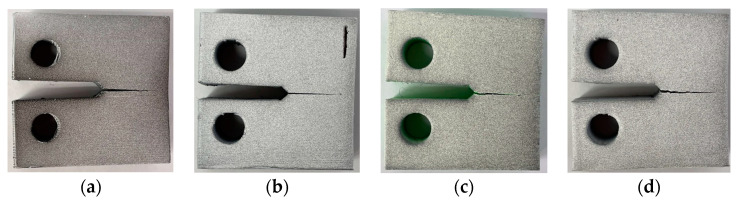
Fractured CT samples (Type D) made of various materials: (**a**) ABS, (**b**) ABS + CF, (**c**) ABS + GF, and (**d**) ABS + BF.

The images of the fractured CT samples are shown in Figure 6. It is visible that crack propagation in these specimens occurs sequentially along the layers, which confirms weak interfacial contact. The cracks also showed a high likelihood of advancing through the layer-to-layer interface, penetrating the printing layer and continuing its growth between another pair of layers.

### 3.2. Bending Tests

**Figure 7:**
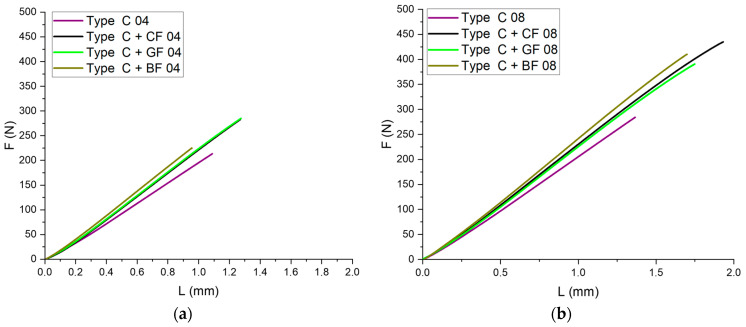
Force–displacement dependences for the series of Type C specimens manufactured with nozzles of (**a**) 0.4 mm and (**b**) 0.8 mm diameter.

The influence of layer adhesion on the strength of samples can be determined by conducting three-point bending tests. For this purpose, Type C specimens were investigated. The values of bending strength and bending modulus, grouped by the SF type and nozzle diameter, are provided in Table 4. The force–displacement curves obtained from bending tests are shown in Figure 7. All printed samples demonstrated close-to-linear elastic deformation.

### 3.3. Analysis of Microstructure 

**Figure 8:**
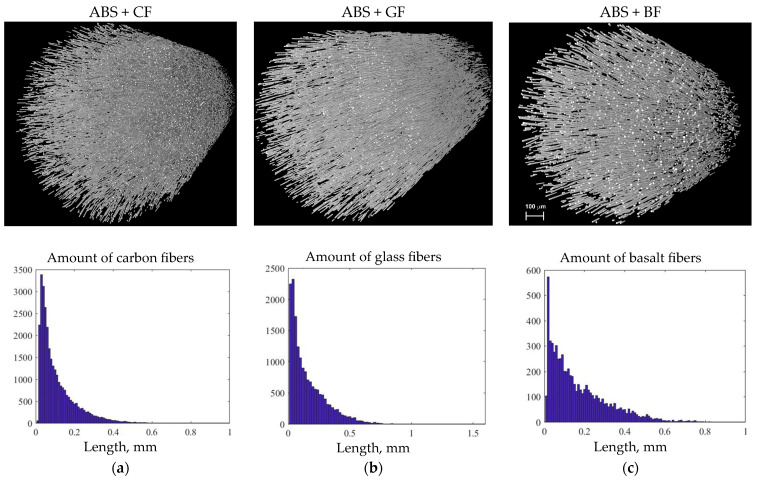
Micro-CT images and histograms of fiber length distribution in filaments: (**a**) ABS + CF, (**b**) ABS + GF, and (**c**) ABS + BF.

Figure 8 shows the histogram of SF length distribution in all types of the filament samples obtained using a statistical analysis of the micro-CT images.

The volume content of SF in filaments, estimated from the micro-CT 3D model, was near 3.5% for all studied samples. Statistical analysis showed that the average diameter of short CF was 8.2 µm, with a predominant length ranging from 10 to 50 µm. However, individual fibers longer than 400 µm were also found in the filament. The glass fibers had a diameter of 14.2 µm, with a predominant length between 10 and 50 µm, but instances of lengths up to 1 mm were also observed. Basalt fibers, on the other hand, had lengths ranging from 10 to 70 µm, including individual fibers up to 800 µm in length. The average diameter for basalt fibers was 15.18 µm.

### 3.4. Analysis of Fracture Surface 

**Figure 9:**
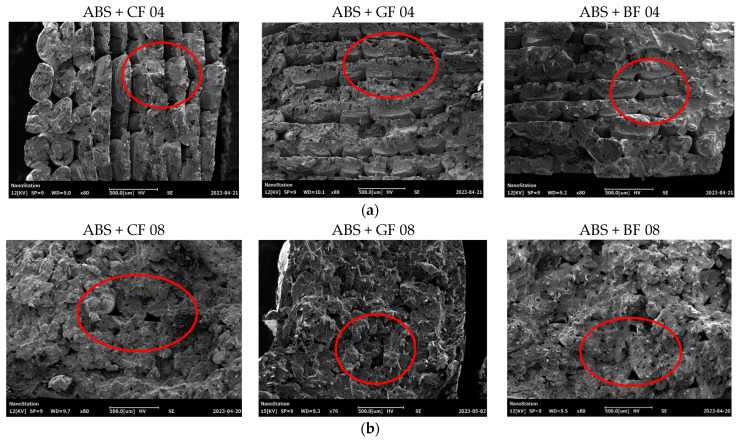
SEM images of fracture surface of Type A samples manufactured using different nozzles: (**a**) 0.4 mm and (**b**) 0.8 mm.

A morphological analysis of the fractured surfaces was conducted for Type A samples after performing uniaxial tensile tests. Figure 9 shows images of samples printed with 0.4 mm and 0.8 mm nozzles obtained using a scanning electron microscope (SEM). The images were taken from the normal to the fracture surface. The defects in the form of voids at the boundaries of the layers are highlighted in red.

**Figure 10:**
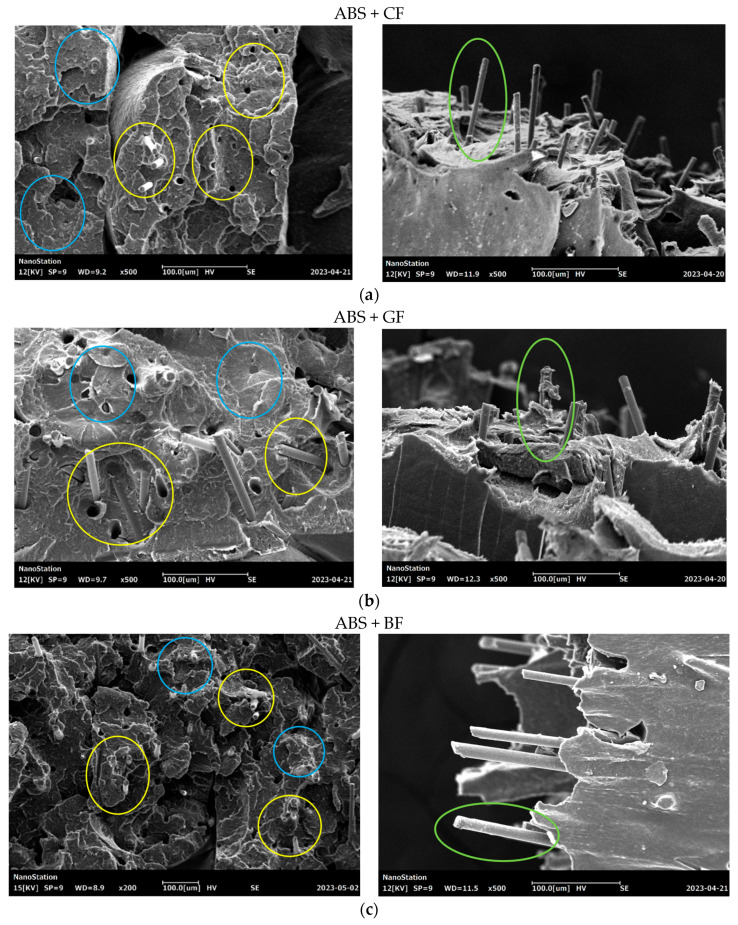
Different scenarios of fiber fracture in Type A samples and images of pulled-out fibers: (**a**) ABS + CF, (**b**) ABS + GF, and (**c**) ABS + BF.

The behavior of SF in specimens during fracture corresponds to one of two main scenarios: (1) fibers break on the fracture surface of the specimen, and (2) fibers are pulled out from the polymeric matrix, leaving voids. Typical cases of short-fiber failure that were captured for the studied samples are demonstrated in Figure 10. Areas where fibers were fractured, according to the first scenario, are highlighted in blue. The areas highlighted in yellow indicate those fibers that fail, corresponding to the second scenario. The voids were formed by the extraction of fibers from the polymer matrix which can be observed, along with visible fractured reinforcing fibers.

**Figure 11:**
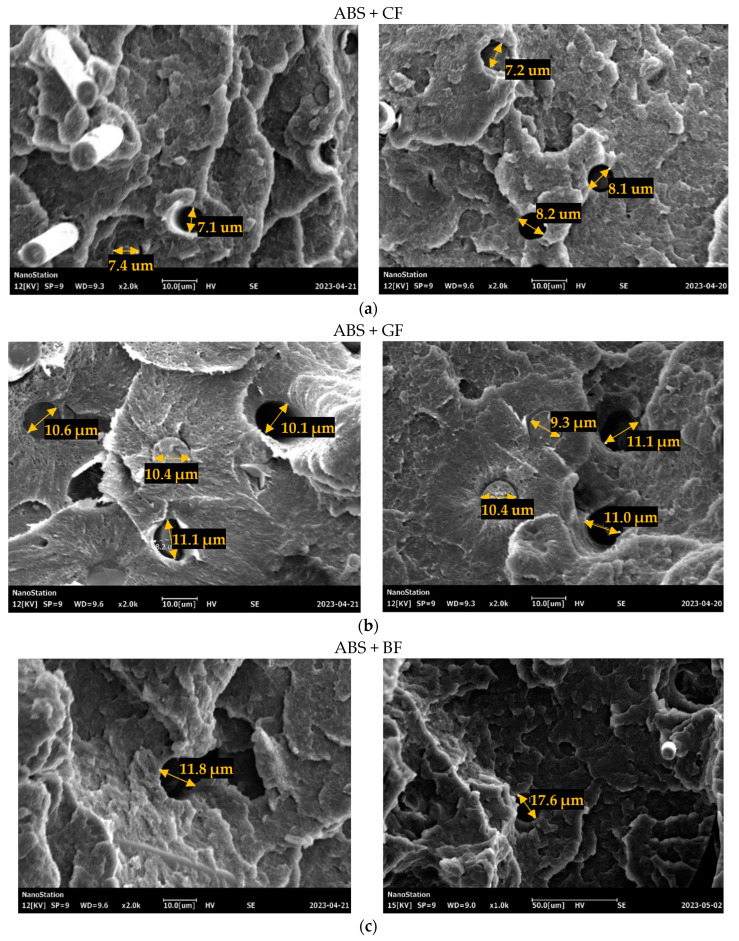
Typical diameters of voids and fibers in Type A specimens after fracture: (**a**) ABS + CF, (**b**) ABS + GF, and (**c**) ABS + BF.

Based on the results of the morphological analysis of the specimen surfaces (and as confirmed by the results of the micro-CT analysis), the average fiber diameter was 8.2 μm for CF, 14.15 μm for GF, and 15.18 μm for BF (see Figure 11). At the same time, the average diameter of voids was 7.8 μm for CF, 10.55 μm for GF, and 15.2 μm for BF. Therefore, the voids formed during SF extraction had a smaller or equal average diameter compared to the fiber diameter itself, indicating the presence of compressive stresses acting in the matrix on the fiber, with the most significant average “void diameter—fiber diameter” ratio observed for ABS + GF specimens.

## 4. Discussion

It was shown that 3D printing using ABS filaments reinforced with CF, GF, or BF can significantly improve the mechanical properties of the specimens in the case of the infill angle oriented along the axis of applied load. The highest values of tensile strength and modulus of elasticity were recorded for the specimens filled with CF. In particular, the combination of CF reinforcement and a 0.8 mm diameter nozzle yielded a strength increase of 60% compared to pure ABS, while reinforcement with GF and BF increased the strength limits by 55% and 58%, respectively. Moreover, the addition of CF increased the modulus of elasticity by 1.75, GF by 1.59, and BF by 1.6 compared to pure ABS. In cases when load application was not aligned with the infill angle, SF reinforcement did not significantly improve the tensile strength limit.

**Figure 12:**
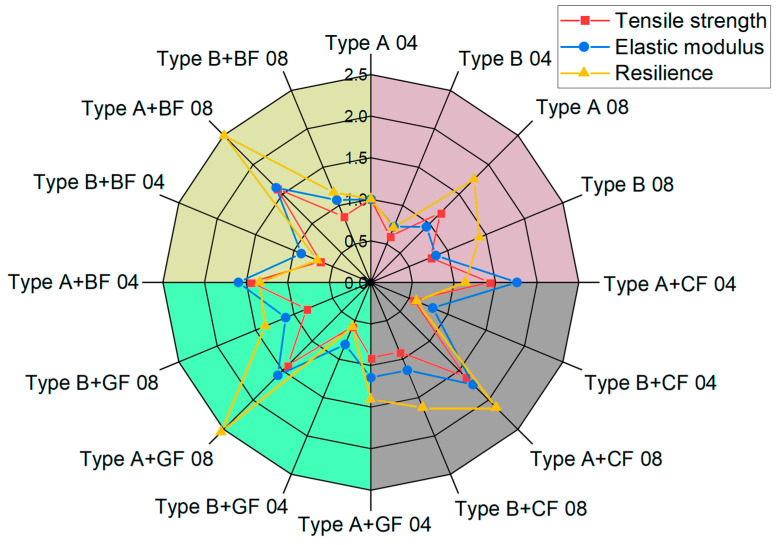
Tensile strength, elastic modulus, and resilience of pure ABS and reinforced ABS obtained for Type A and Type B samples using nozzles of different diameters.

The spider web chart, where tensile strength, elastic modulus, and resilience values for all the studied samples are normalized by the corresponding values obtained for the pure ABS (Type A 04 sample: infill angle 0°, 04 mm nozzle), is shown in Figure 12.

The maximum strength (79.12 ± 1.65 MPa) was achieved for samples with CF reinforcement printed with a nozzle of 0.8 mm diameter and a 0° infill angle (Type A + CF 08). This is a 36% increase in the strength limit compared to pure ABS. Similarly, the reinforcement of ABS with CF resulted in the highest modulus of elasticity of 9.24 GPa for samples printed with a 0.4 mm nozzle diameter (Type A + CF 04). The addition of GF and BF also showed almost the same increase in the strength limit (31% for GF and 34% for BF). However, for the specimens for which the infill angle is not aligned with the applied force (Type B), the strength limit was reduced by 1.85 times compared to the infill angle oriented in the direction of applied forces. However, taking into account the obtained resilience values, all types of reinforcement extended the ability of the 3D-printed material to absorb energy during elastic deformation. The greatest increase, by more than 45%, was observed for samples with GF reinforcement, slightly less, about 42%, was observed for those with BF, and the lowest was observed for specimens with CF—just over 21%.

Samples with CF reinforcement demonstrated superior fracture toughness properties compared to the ABS with GF and BF, as well as in comparison to pure ABS. The obtained results showed that among all the considered fillers, only the addition of CF had a notable impact on the increase in fracture toughness properties, while thew results for ABS with the addition of GF and BF were close and even slightly less than that of the pure ABS. As can be seen from the images in Table 3, the crack propagation lengths and surrounding local strain fields at a force of 490 N were different for all considered CT samples. The lowest deformation for crack propagation was required for ABS without filler. It can also be noted that the crack propagation rate depended on the type of SF filler.

**Figure 13:**
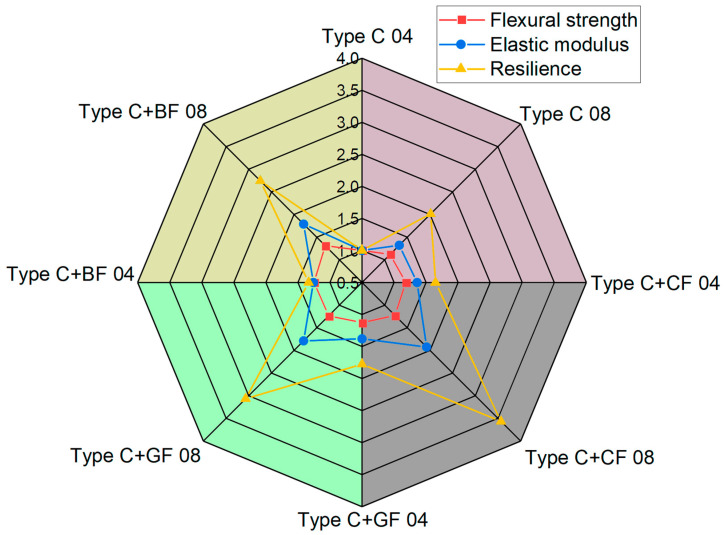
Comparison of flexural strength and modulus of elasticity for pure ABS and SF-reinforced ABS printed with nozzles of different diameters.

The spider web chart for normalized values of flexural strength, elastic modulus, and resilience obtained in bending tests is displayed in Figure 13.

The maximum bending strength was achieved using a 0.8 mm diameter nozzle for all types of SF fillers. The highest bending strength of 29.47 ± 1.16 MPa was observed for ABS + CF samples (Type C + CF 08). The maximum bending modulus of 1734.62 ± 58.36 MPa was achieved for ABS + BF, also manufactured using a 0.8 mm diameter nozzle. In general, samples printed with a 0.8 mm diameter nozzle showed higher deformation compared to those printed with a 0.4 mm diameter nozzle. However, SF reinforcement does not address the issue related to the nozzle diameter’s impact on the interlayer bonding strength of the specimens. In the case of pure ABS, the difference between results for a sample printed with 0.4 mm and 0.8 mm diameter nozzles was 24.4%, while for BF it was 29.7%, 23% for GF, and 29.4% for CF reinforcement.

All SF-reinforced samples demonstrated a brittle type of fracture. By comparing specimens manufactured with nozzles of different diameters, it was observed that using a 0.4 mm nozzle resulted in more layers being deposited with a thinner thickness, leading to an increase in the number of voids and zones of increased stress at the boundaries of the deposited material. Considering the same manufacturing settings, such as printing speed and temperature, as well as layer boundary sharpness, it can be concluded that interlayer adhesion was better for samples printed with a 0.8 mm diameter nozzle. Differences in types of SF filler do not affect the formation of voids and interlayer adhesion, as is confirmed by a microscopic analysis of specimen cross-sections. Their structure thus remained identical.

The distribution of SF in polymers is also a crucial factor that influences the resulting mechanical properties. An optimal fiber length’s distribution ensures uniform reinforcement, improving performance. The fiber length, as well as diameters of fibers, determines the area of contact surfaces between the fibers and the matrix, affecting the adhesion properties. According to the SEM images (see Figure 10), residues of the matrix can be directly observed on the pulled-out fibers, indicating the degree of adhesion between the fiber and the polymer matrix; the greater the residue of matrix material on the fiber, the higher the degree of adhesion and the greater the force required to pull the fiber out of the matrix. Some specifics connected to the type of short fiber can be also noted. The highest ratio between the average fiber pulled-out length to the fiber’s diameter was recorded for the ABS+CF composites, which indicated lower adhesion properties. According to the microscopic analysis of the void’s diameter, GF-reinforced samples demonstrated the highest level of residual stress in the matrix.

## 5. Conclusions

This study investigated the influence of reinforcing fillers such as short CF, GF, and BF on the characteristics of ABS, one of the most used thermoplastic materials for 3D printing. Mechanical tests were conducted using standard-type samples. The obtained results were compared based on the type of filler and the variation in the manufacturing parameters.

The results demonstrated the possibility to control and improve the properties of 3D-printed ABS parts by using a filament with the addition of SF. The incorporation of this filler leads to several advantages, such as an increase in elastic modulus and tensile strength as well as fracture toughness. The nozzle diameter was one of the most important parameters that affected the mechanical characteristics of samples during tensile and bending tests. The use of a larger diameter nozzle resulted in an increase in the layer’s width, allowing to even out the stress distribution in each printed path of the structure and reduce zones of stress concentration. However, other factors such as temperature fluctuations and thermal transfer between the layers were not considered during the experiment in this study and may also influence the mechanical characteristics. Some mechanical properties were not affected by the addition of the SF when using specific combinations of the applied load and printing parameters. Maximization of the positive effect could be achieved when the infill angle and printing direction were aligned with the direction of the applied load.

Despite promising advantages, the addition of an SF into ABS for 3D printing has certain challenges. These include achieving the uniform dispersion of fibers within the filament, optimizing printing parameters to minimize nozzle clogging, and maintaining dimensional accuracy and a surface finish. Additionally, there may be concerns regarding the wear and tear of printing nozzles due to the abrasive nature of some SF materials. Future endeavors are aimed at refining manufacturing processes, optimizing fiber–matrix interactions, and exploring novel fiber materials to further enhance the performance and versatility of 3D-printed parts. 

## Figures and Tables

**Table 1:** Mechanical properties of specimens under tensile testing.

	Elastic Modulus (GPa)	Tensile Strength (MPa)	Resilience (J*m^−3^)
**Type A 04**	5.26±0.10	49.22±1.66	132.99±12.90
**Type B 04**	3.82±0.17	29.43±2.14	94.63±17.66
**Type A 08**	4.98±0.30	58.14±3.38	233.41±56.67
**Type B 08**	4.46±0.43	38.32±4.41	187.83±44.50
**Type A + CF 04**	9.24±0.16	70.14±2.61	151.04±15.45
**Type B + CF 04**	4.23±0.25	27.36±2.87	78.49±22.21
**Type A + CF 08**	9.14±0.20	79.12±1.65	283.22±49.66
**Type B + CF 08**	6.02±0.40	44.62±3.12	216.58±23.96
**Type A + GF 04**	8.31±0.21	69.63±2.62	187.30±19.71
**Type B + GF 04**	4.26±0.41	28.36±3.72	77.04±13.61
**Type A + GF 08**	8.37±0.26	76.31±2.53	338.84±56.69
**Type B + GF 08**	5.83±0.51	41.11±3.17	182.84±16.06
**Type A + BF 04**	8.38±0.18	70.98±1.81	177.74±5.34
**Type B + BF 04**	4.76±0.36	32.50±2.95	92.62±12.38
**Type A + BF 08**	8.47±0.11	78.25±1.30	331.62±20.71
**Type B + BF 08**	5.64±0.47	42.19±2.69	155.91±17.88

**Table 2:** Results of tensile testing of compact tension specimens.

	$K_{Ic},MPa*\sqrt{m}$	$G_{Ic},\frac{kJ}{m^{2}}$	Strength (MPa)
**ABS**	1.28	0.031	0.62
**ABS + CF**	1.65	0.046	0.77
**ABS + GF**	1.25	0.030	0.58
**ABS + BF**	1.28	0.029	0.59

**Table 3:** Strain fields at the surface of Type D samples obtained using the VIC-3D system at the initiation of crack growth and crack opening at a load value of 490 N.

	ABS	ABS + CF	ABS + GF	ABS + BF
Initiation of crack growth	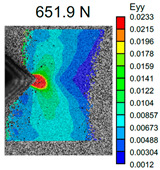	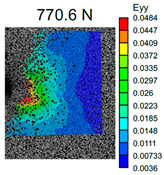	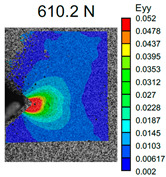	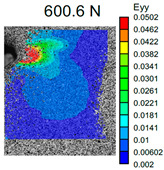
Crack opening	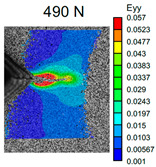	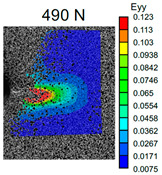	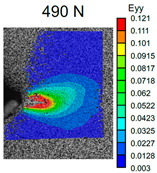	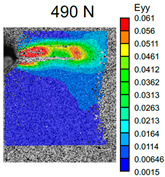

**Table 4:** Flexural mechanical properties obtained in three-point bending tests.

	Bending Modulus (GPa)	Bending Strength (MPa)	Resilience (J*m^−3^)
**Type C 04**	1.32±0.03	15.30±1.77	8.38±1.84
**Type C 08**	1.48±0.01	20.23±1.47	16.87±2.05
**Type C + CF 04**	1.56±0.03	19.26±2.27	13.80±2.37
**Type C + CF 08**	1.62±0.05	27.38±1.39	29.88±2.54
**Type C + GF 04**	1.49±0.03	21.09±1.59	14.87±2.15
**Type C + GF 08**	1.63±0.06	27.39±1.27	25.68±2.32
**Type C + BF 04**	1.65±0.07	20.80±1.72	11.17±2.53
**Type C + BF 08**	1.73±0.06	29.47±1.16	22.98±2.07

## Data Availability

The raw data supporting the conclusions of this article will be made available by the authors on request.
